# Serum Calprotectin: An Antimicrobial Peptide as a New Marker For the Diagnosis of Sepsis in Very Low Birth Weight Newborns

**DOI:** 10.1155/2011/291085

**Published:** 2011-05-30

**Authors:** Gianluca Terrin, Annalisa Passariello, Francesco Manguso, Gennaro Salvia, Luciano Rapacciuolo, Francesco Messina, Francesco Raimondi, Roberto Berni Canani

**Affiliations:** ^1^Department of Women Health and Territorial Medicine, University of Rome “La Sapienza”, 00185 Rome, Italy; ^2^Department of Pediatrics, University of Naples “Federico II”, 80131 Naples, Italy; ^3^Neonatal Unit, “V. Monaldi” Hospital, 80131 Naples, Italy; ^4^A. Cardarelli Hospital, 80131 Naples, Italy; ^5^Neonatal Unit, “Fatebenefratelli” Hospital, 80123 Naples, Italy; ^6^Neonatal Unit, “Villa Betania” Evangelical Hospital, 80100 Naples, Italy

## Abstract

To determine the diagnostic utility of serum calprotectin, a mediator of innate immune response against infections, we performed a multicenter study involving newborns with a birth weight <1500 g and a postnatal age >72 hours of life. The diagnostic accuracy of serum calprotectin was compared with that of the most commonly used markers of neonatal sepsis (white blood cell count, immature-to-total-neutrophil ratio, platelet count, and C-reactive protein). We found that the serum calprotectin concentration was significantly higher (*P* < .001) in 62 newborns with confirmed sepsis (3.1 ± 1.0 *μ*g/mL) than in either 29 noninfected subjects (1.1 ± 0.3 *μ*g/ml) or 110 healthy controls (0.91 ± 0.58 *μ*g/ml). The diagnostic accuracy of serum calprotectin was greater (sensitivity 89%, specificity 96%) than that of the traditional markers of sepsis. In conclusion, serum calprotectin is an accurate marker of sepsis in very low birth weight newborns.

## 1. Introduction

The neonatal immune response to many pathogens is largely immature [1]. At this age, infections are characterized by a high mortality and morbidity [2, 3]. An early diagnosis is crucial because the clinical course may be fulminating, particularly in very low birth weight (VLBW) babies, in whom the onset is often inconspicuous, with minimal, subtle, and nonspecific signs [1–3]. Isolation of bacteria from central body fluid (usually blood) is the standard test for neonatal systemic infection; however, the result of culture is not available before 24–48 h and is negative in many instances, even in cases of a clear clinical picture of sepsis [4–6]. 

Neonatal septicaemia is associated with hyperinflammatory host responses that subtend activation of immune system. A broad spectrum of inflammatory markers has been proposed for the diagnosis of neonatal sepsis [4]. However, most of these markers are mediators of an acquired immunity response, which is largely immature in the neonatal period [4]. On the contrary, innate immunity is fully developed in the first weeks of life, but the potential diagnostic role of components of innate immunity has not been investigated [7, 8].

Calprotectin (aka MRP8/14, calgranulin, cystic fibrosis-associated antigen, and S100), a major product of innate immunity cells, is an antimicrobial peptide that protects cells against invasive microorganisms and regulates adhesion of leukocytes to the endothelium and extracellular matrix during the inflammatory process [4, 9]. Calprotectin is released by innate immunity cells immediately after host-pathogen interaction [9] and is detectable in body fluids by means of a simple ELISA technique [10, 11].

Calprotectin has been proposed for the diagnosis of many inflammatory conditions [9]. However, its use in the diagnosis of neonatal sepsis remains unexplored. The aim of this study was to investigate the diagnostic power of serum calprotectin (SC) measurement in VLBW infants with sepsis. 

## 2. Materials and Methods

This prospective investigation involved 3 Neonatal Intensive Care Units (NICUs) in the south of Italy over a 24-month enrolment study period. The study protocol was approved by the Ethics Committee of each center. Written informed consent was obtained from the parents of all infants enrolled in the study. The research was not sponsored by any company. 

Newborns were eligible for the study when they fulfilled all of the following criteria: (1) gestational age > 24 weeks; (2) birth body weight (BW) < 1500 g; (3) postnatal age > 72 hours; (4) no antibiotic treatment for at least 2 weeks before enrolment; (5) parental consent. Infants with the following conditions were excluded: (1) Apgar score < 3 at 5 min; (2) critical clinical conditions, as indicated by a blood pH < 6.8, or by the presence of hypoxia with persistent bradycardia; (3) incomplete clinical data or deviation from the study protocol; (4) maternal history of immunologic, inflammatory, or infectious diseases requiring antibiotic therapy during pregnancy, including the labour/delivery process; (5) surgery; (6) intraventricular haemorrhage grades III-IV [12]. Enrolled subjects were prospectively divided into 2 groups according to the presence or absence of features suggestive of sepsis. 

According to standardized criteria [4, 5, 13, 14], systemic infection was suspected in the presence of ≥ 2 of the following clinical features: poor perfusion (capillary refilling > 3 seconds), muscle hypotonia or hypertonia, lethargy, enteral nutrition intolerance, bloody stools, progressive increase in O_2_ requirement, bradycardia, unstable body temperature, unexplained and persistent metabolic acidosis (base deficit > 10 mmol/L), and/or hyperglycaemia (>10 mmol/L). White blood cell count (WBC), immature-to-total-neutrophil (I/T) ratio, platelet count, serum C-reactive protein (CRP), cultures of blood, urine, and endotracheal aspirate (when patient was intubated) for bacteria and fungi, chest or abdominal radiograph if the patient presented signs suggestive of thoracic or intra-abdominal disease, cardiac and brain ultrasonography, electroencephalography (EEG), and screening tests for metabolic diseases were performed in all subjects with suspected sepsis. 

According to previously standardized criteria [5, 13, 15, 16] we divided the patients into (1) a septic group, constituted by infants with suspected episodes of sepsis that had been confirmed as systemic infection by positive cultures (culture-proven sepsis) or by the presence of a typical clinical picture of sepsis associated with at least one positive traditional laboratory marker of infection and a clear improvement after a full course of antibiotic therapy (high probable sepsis); (2) a nonseptic group, constituted by infants who met the initial criteria for suspected sepsis but were subsequently classified as being noninfected and had a definitive diagnosis unrelated to sepsis, or of neonates in whom symptoms disappeared without antibiotic therapy within 8 hours. 

Blood samples for SC determination were collected within 60 min of the onset of symptoms. For comparison, blood specimens were collected during routine sampling to determine SC levels from newborns without symptoms of sepsis, that is, the control group. The results of SC measurements were concealed until statistical analysis was concluded.

### 2.1. Laboratory Procedures

All laboratory investigations were performed by researchers unaware of the clinical data. Complete blood counts were done by cell counter [17]. Leukocytes were differentiated by microscopy. The I/T ratio was calculated as the sum of immature granulocytes divided by the sum of all neutrophils [17]. The CRP was measured by rate nephelometry [14]. Serum calprotectin was measured by a commercial ELISA assay (Calprest, Eurospital, Trieste, Italy) [9–11]. In brief, a blood sample (0.5 mL) for serum calprotectin measurements was collected in blood collection tubes containing ethylenediaminotetraacetic acid (EDTA). The sample was centrifuged for 10 min at 10000 rpm and the extracted serum was collected and frozen at −20°C for subsequent measurement. The serum was diluted 1 : 50, and 100 *μ*L of each sample was added to the wells of a plate and incubated at room temperature for 45 min. The plate was then washed 3 times with diluted washing solution, and 100 *μ*L of purified rabbit anticalprotectin antibodies conjugated with alkaline phosphatase were added and incubated for 45 min at room temperature. A second washing procedure was performed, 100 *μ*L of enzyme substrate solution was added to each well, and optical density was read at 405 nm. Serum calprotectin concentration was calculated from the standards and expressed as *μ*g/mL [9–11]. The minimum concentration of human calprotectin detected with this kit is 1.6 ng/mL. 

### 2.2. Statistical Analysis

The Pearson chi-square test was performed in the case of categorical variables. Continuous variables were expressed as mean and 95% confidence (CI). The groups were compared for variables by the one-way ANOVA procedure with the Bonferroni test for the posthoc analysis. After checking for assumptions, linear regression analysis with a stepwise method was used to study the possible effect of the different variables (BW, gestational age, sex, age at enrolment, mode of delivery, prenatal steroid use, WBC, site of infection and pathogen involved, presence of respiratory distress syndrome, patent ductus arteriosus, and periventricular leukomalacia) on SC concentration. Binary logistic regression analysis was performed using as dependent variable the presence of sepsis and as independent variables WBC, I/T ratio, platelet count, CRP, and SC. 

All tests of significance were two sided. A *P* value of ≤.05 was considered significant. The optimal cut-off SC value to distinguish septic from nonseptic patients among subjects investigated for suspected sepsis was determined using receiving operating characteristic (ROC) curve analysis. Statistical analysis was performed using the SPSS software package for Windows (release 16.0.0; SPSS Inc., Chicago, Ill, USA) and StatsDirect (release 2.6.6).

## 3. Results

A total of 231 newborns were eligible for the study. Twenty-eight were excluded because of incomplete clinical data or deviation from the study protocol (10 patients), intraventricular haemorrhage (8 patients), critical clinical condition (8 patients), and lack of parental consent (2 patients). Ninety-three newborns (35, 32, and 26, per Center) were investigated for suspected sepsis: 62 were classified as “septic” (52 culture proven: *12 E. coli,* 8 *Enterococcus faecalis, *6 *Pseudomonas aeruginosa*, 6 *Klebsiella pneumonia, *6 *Staphylococcus coagulase negative, *6 *Candida albicans, 5 Serratia marcescens,* 3* Streptococcu*s *pyogenes*, and 10 high probable sepsis), 29 were included in nonseptic group, and two infants were lost to followup (transferred to other hospitals). One hundred and ten infants were enrolled as controls. The main clinical and demographic characteristics were similar in the three groups (Table 1).

**Figure 1:**
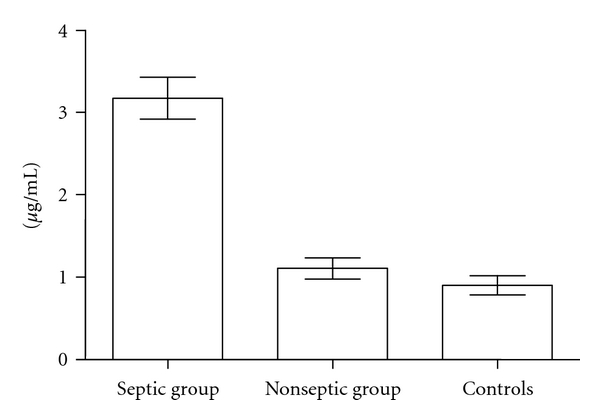
Levels of serum calprotectin in septic, nonseptic, and control VLBW newborns.

Serum calprotectin concentration was higher in the septic group than in the nonseptic and control groups (Figure 1). Value of CRP was significantly different between septic and control group but not between septic and nonseptic patients (Table 2). The linear regression analysis demonstrated that SC was not influenced by BW, gestational age, sex, age at the enrolment, mode of delivery, prenatal steroid use, WBC count, site of infection and the offending pathogen, presence of respiratory distress syndrome, patent ductus arteriosus, and periventricular leukomalacia. 

**Figure 2:**
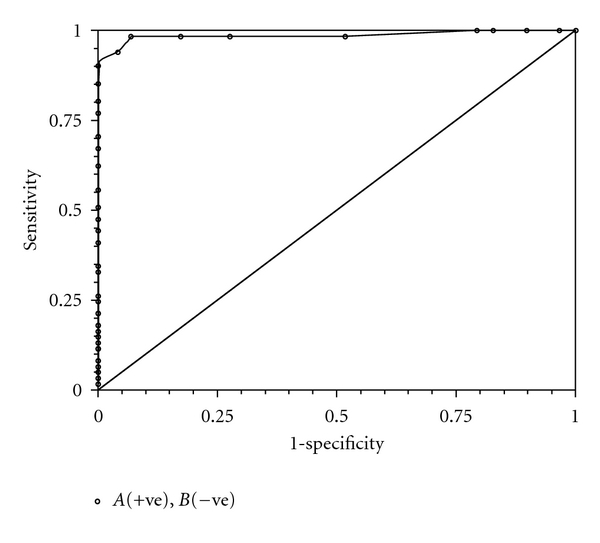
Receiver operating characteristic curve defining an optimal serum calprotectin cut-off value (1.7 *μ*g/mL) to distinguish septic from nonseptic symptomatic patients.

The optimal cut-off serum calprotectin value to distinguish infants with sepsis from infants without sepsis was 1.7 *μ*g/mL as determined by the ROC curve analysis, characterized by a vast area under curve demonstrating an excellent discrimination power (Figure 2). At this cut-off value, all infants with fungi-related sepsis (*n* = 6) had SC levels above normal value, whereas only 2/6 had deranged values of traditional markers (1 with platelet count >500.0 × 10^3^/mm^3^; 1 with CRP >10 mg/dL). The diagnostic accuracy of all tests evaluated is reported in Table 3. Binary logistic regression analysis showed that SC (OR 164.6; 95% CI 21.6–1255.1; *P* < .001) and CRP (OR 1.1; 95% CI 1.0-1.1; *P* = .007) were predictors of sepsis in our population. 

## 4. Discussion

A high sensitivity and negative predictive value, associated with a good specificity and positive predictive value, supports the use of SC in the diagnosis of sepsis in a VLBW newborn. 

A limited number of pathogens are responsible for sepsis among neonates. Most pathogens are encapsulated bacteria [1]. The immunologic advantage of this capsule is that the bacteria elude phagocytic killing, because the coating blocks complement binding and opsonization. 

Acute phase proteins, such as CRP [4], and the production of antibodies against the polysaccharide promote the elimination of the pathogens [4]. However, this immune response takes precious time [1], while antimicrobial peptides are promptly and abundantly released after the host-pathogen interaction [4, 8, 9].

In this context, SC measurement could overcome many of the limitations of the most common markers proposed for the diagnosis of neonatal sepsis, that is, haematological tests, acute phase proteins, chemokines, cytokines, and adhesion molecules [4]. Haematological markers have a low accuracy; moreover, sensitivity and specificity vary widely across studies being between 17% and 90% and 31% and 100%, respectively [4, 5, 18, 19]. In our study, WBC count did not differ significantly between septic and nonseptic VLBW subjects. Differently, the I/T ratio was higher in septic patients than in either nonseptic or control subjects. Unfortunately, the low sensitivity of this marker limits its use in clinical practice. The most widely used acute phase reactant for the diagnosis of neonatal sepsis is CRP [4, 18]. However, because the concentration of CRP increases rather slowly in the initial phase of inflammatory response to pathogens, the sensitivity of CRP testing at the time of sepsis evaluation is insufficient [4]. Our results confirm this observation. Moreover, CRP is elevated only in a minority of infants affected by fungi-related sepsis [4]. Another acute phase marker is procalcitonin (PCT). Its diagnostic profile has been claimed to be superior to other acute phase proteins, including CRP. However, serum PCT concentrations are variable in the first few days of life, and very high serum concentrations of PCT have been detected in patients with acute lung and inhalation injuries and cardiac diseases, without evidence of infection [4]. Chemokines, cytokines, and adhesion molecules have a high diagnostic accuracy for sepsis, but the lack of adequate technology in many neonatal intensive care units and high costs have impeded the widespread use of these markers in clinical practice [4, 14, 15, 19]. More recently, a proteomic quantitative approach has been proposed to identify biomarkers for the diagnosis of sepsis [20]. Despite promising results, the application of proteomic techniques is not feasible in hospital laboratories that lack specific facilities. Another limitation of the proposed proteomic protocol may not differentiate between proteins that occur in low concentrations in plasma and that have a similar molecular weight, namely, interleukins, antimicrobial peptides, and chemokines.

We found that SC is an early, accurate, and easy-to-use marker of neonatal sepsis. When measured upon the appearance of the first clinical signs of sepsis, its sensitivity, PPV, and NPV were higher than those reported for traditional tests [2–4]. Serum calprotectin levels were not influenced by postnatal age, which is another advantage over some traditional diagnostic tools [4]. The ELISA technique used to detect SC is simple, rapid, and inexpensive and requires only a small volume of blood sample thereby reducing the risk of iatrogenic anaemia [11]. Moreover, the ELISA technique for SC is now available in a more rapid and less expensive bed-side test, particularly useful in neonatology [21].

The encouraging results reported herein provide a rationale for investigations of the role of SC in the management of sepsis. In particular, it may be worthwhile to assess the prognostic role of SC as regards the severity of sepsis. The high NPV of SC suggests that it could be an aid in therapy decision-making. In this context, the optimal cut-off value defined in this study could be useful in further research in this field. Finally, SC could help to resolve the dilemma of whether to start antibiotics immediately after sepsis screening and when to stop treatment, but there is a need for studies specifically designed to address these issues. 

The study has some limitations. Firstly, as reported in other studies [4, 5, 13–16], some of the patients that we diagnosed with sepsis had negative microbiological examinations. Following previous studies, we classified such cases as “high probable sepsis”. These infants presented the typical clinical picture of sepsis associated with positive traditional laboratory markers of infection. All patients underwent a clear clinical improvement after a full course of antibiotic treatment. Thus, it is unlikely that we misclassified these patients. Secondly, we studied only VLBW infants and excluded asphyxia and critical ill subjects. Further research is needed to investigate the diagnostic accuracy of SC in these settings.

To conclude, ours is the first study to investigate an innate immunity component as a biomarker of septicaemia in VLBW infants. Serum calprotectin could be a practical marker in the diagnostic approach to VLBW infants with suspected sepsis, and it could open the way to investigations exploring the role of other components of innate immunity in the diagnostic workup of neonates with infectious conditions.

##  Conflict of Interests

The authors declare that there is no conflict of interests.

## Figures and Tables

**Table 1:** Main demographic and clinical characteristics of the study population.

	Septic group	Nonseptic group	Controls
No.	62	29	110
Birth weight, g	1082 (1029–1135)	1178 (1080–1275)	1088 (1045–1131)
Gestational age, weeks	29.2 (28.7–29.9)	30.1 (29.1–31.1)	29.2 (28.7–29.6)
Male, *n* (%)	39 (63)	13 (45)	67 (61)
Spontaneous delivery, *n* (%)	10 (16)	3 (11)	13 (12)
Intubation for respiratory distress syndrome, *n* (%)	53 (85)*	11 (38)	34 (31)
Duration of invasive mechanical ventilation before enrolment	5.8 (6.5–9.7)	4.8 (2.5–7.0)	5.9 (4.5–7.2)
Subjects with central vascular access, *n* (%)	30 (48)	12 (41)	36 (33)
Age at enrolment, days	8.7 (7.2–10.1)	6.8 (5.5–8.0)	6.9 (6.1–7.7)

Data expressed as mean (95% Confidence Interval for Mean) if not specified.

*P < .001 versus other two groups.

**Table 2:** Results of laboratory parameters evaluated.

	Septic group	Nonseptic group	Controls		*P* values	
				Septic versus nonseptic	Septic versus controls	Nonseptic versus controls
White blood cell count (*n* × 10^3^/mm^3^)	16.645 (12.219–21.098)	14.837 (11.619–18.055)	12.382 (10.688–14.076)	1.000	.106	1.000
I/T ratio	0.91 (0.58–1.24)	0.030 (0.01–0.06)	0.042 (0.02–0.06)	.025	.025	1.000
Platelet count (*n* × 10^3^/mm^3^)	278 (239–317)	289 (256–322)	259 (237–281)	1.000	1.000	.802
C-reactive protein (mg/dL)	13.1 (7.4–18.7)	6.2 (0.38–12.8)	2.1 (1.7–2.6)	.123	<.001	.591
Serum calprotectin (*μ*g/mL)	3.1 (2.9–3.4)	1.1 (0.9–1.2)	0.91 (0.8–1.0)	<.001	<.001	.515

I/T ratio: immature-to-total-neutrophil ratio.

Data expressed as mean (95% Confidence Interval for Mean) if not specified.

**Table 3:** Diagnostic accuracy of various markers in identifying septic patients among symptomatic newborns.

	Sensitivity	Specificity	PPV	NPV	Likelihood ratio (+ test)
WBC	14 (7–26)	93 (77–99)	82 (48–98)	34 (23–45)	2 (1–8)
I/T ratio	60 (46–72)	82 (64–94)	88 (74–96)	48 (34–64)	3 (2–8)
Platelet count	13 (6–24)	76 (56–90)	53 (27–79)	29 (19–40)	0.5 (0-1)
CRP	29 (18–42)	93 (77–99)	90 (68–99)	38 (27–50)	4 (1–16)
Serum calprotectin	89 (78–95)	96 (82–99)	98 (90–99)	80 (63–92)	26 (5–145)

PPV: positive predictive value. NPV: negative predictive value. WBC: white blood count. CRP: C-reactive protein. I/T ratio: immature-to-total-neutrophil ratio.

N.B. Cut-off value: total neutrophil count < 4.0 or > 25.0 ×  10^3^/mm^3^; platelet count < 50.0 or > 500.0 × 10^3^/mm^3^; I/T ratio > 0.2; CRP >10 mg/dL; serum calprotectin >1.7 *μ*g/mL [8–10, 12, 16].
